# High prevalence of *Trichinella pseudospiralis* in Florida panthers (*Puma concolor coryi*)

**DOI:** 10.1186/s13071-015-0674-z

**Published:** 2015-02-04

**Authors:** Mason V Reichard, Marc Criffield, Jennifer E Thomas, Jacqueline M Paritte, Mark Cunningham, Dave Onorato, Kenneth Logan, Maria Interisano, Gianluca Marucci, Edoardo Pozio

**Affiliations:** Department of Veterinary Pathobiology, Center for Veterinary Health Sciences, Oklahoma State University, 250 McElroy Hall, Stillwater, OK 74078 USA; Fish and Wildlife Research Institute, Florida Fish and Wildlife Conservation Commission, 298 Sabal Palm Rd, Naples, FL 34114 USA; Fish and Wildlife Research Institute, Florida Fish and Wildlife Conservation Commission, 1105 SW Williston Rd, Gainesville, FL 32601 USA; Colorado Parks and Wildlife, 2300 S. Townsend Ave, Montrose, CO 81401 USA; Department of Infectious, Parasitic and Immunomediated Diseases, Istituto Superiore di Sanità, viale Regina Elena 299, Rome, 00161 Italy

**Keywords:** Florida panther, *Puma concolor coryi*, *Trichinella spiralis*, *Trichinella pseudospiralis*, Zoonotic

## Abstract

**Background:**

Parasites of the genus *Trichinella* are zoonotic nematodes common in carnivores throughout the world. We determined the prevalence and species of *Trichinella* infections in Florida panthers (*Puma concolor coryi*).

**Methods:**

Tongues from Florida panthers were collected at necropsy and examined by pepsin-HCl artificial digestion for infection with *Trichinella* spp. DNA was extracted from larvae and multiplex PCR using *Trichinella* species-specific primers was used to genotype the worms.

**Results:**

*Trichinella* spp. larvae were detected in 24 of 112 (21.4%; 14.6%–30.3%) panthers. Sixteen of the panthers (14.3%) were infected with *T. pseudospiralis*, 1 (0.9%) was infected with *T. spiralis*, and 2 (1.8%) had mixed infections of *T. pseudospiralis* and *T. spiralis. Trichinella* spp. larvae from 5 panthers were not identified at the species level due to degraded DNA.

**Conclusions:**

This is the highest prevalence of *T. pseudospiralis* detected in North America up to now and suggests the Florida panther is a key mammalian reservoir of this parasite in southern Florida. *Trichinella pseudospiralis* can infect both mammals and birds indicating the source of infection for Florida panthers could be broader than believed; however, birds represent a small percentage (0.01%) of the cat’s diet. Since wild pigs (*Sus scrofa*) can be parasitized by both *T. pseudospiralis* and *T. spiralis* and these swine can comprise a large portion (~40%) of a panther’s diet in Florida, we believe that Florida panthers acquired these zoonotic parasites from feeding on wild pigs.

## Background

Infection with *Trichinella* species is common in wild carnivores throughout the world [1]. Transmission of *Trichinella* spp. in wild animals is largely based on predator–prey relationships and scavenger behavior. Hosts are infected with *Trichinella* spp. when they ingest muscle tissues containing infective larvae. There are currently 5 species or genotypes of *Trichinella* known in the United States. *Trichinella spiralis*, *T. murrelli, Trichinella* genotype T6, and *T. nativa* which modify the muscle cell to a nurse cell with a thick collagen capsule, and *T. pseudospiralis* which modifies the muscle cell to a nurse cell without a collagen capsule [2]. *Trichinella spiralis* is found primarily in domestic and wild pigs throughout the world and occasionally infects other animals [1,2]. *Trichinella murrelli* predominately infects wild carnivores in temperate regions of the United States, southern Canada, and possibly Mexico [2]. *Trichinella nativa* and *Trichinella* genotype T6 are freeze-resistant species whose natural hosts are carnivores in northern latitudes. *Trichinella pseudospiralis* has a cosmopolitan distribution, can infect meat-eating mammals and is the only *Trichinella* sp. that has been found to infect birds. All of these species of *Trichinella* are zoonotic [2].

The Florida panther (*Puma concolor coryi*) is an endangered species whose current range is restricted to south Florida, but transient males have been documented as far north as central Georgia [3]. Panther home ranges vary from 435–650 km^2^ for males and 193–396 km^2^ for females with both sexes utilizing wetland forests in the daytime and prairie grasslands at night [4,5]. Analysis of prey items from Florida panther kills and scat showed that wild pigs (*Sus scrofa*), white-tailed deer (*Odocoileus virginianus*), raccoons (*Procyon lotor*), 9-banded armadillos (*Dasypus novemcinctus*), and marsh rabbits (*Sylvilagus palustris*) comprise the majority of the cat’s diet [6,7]. Because Florida panthers are strict carnivores, their risk for being exposed to *Trichinella* spp. is high. Previous analysis of 7 Florida panthers demonstrated that 4 (57.1%) were infected with *Trichinella* sp. [8]. However, in 1985, methodologies necessary to reliably and conveniently distinguish species of *Trichinella* were not available and the first-stage larvae were not identified further. The objectives of the present paper were to reassess the prevalence of *Trichinella* spp. infection in Florida panthers and determine which species of *Trichinella* infect the endangered cats. We report that 24 of 112 (21.4%) Florida panthers were infected with *Trichinella* spp. Sixteen of the panthers were infected with *T. pseudospiralis,* 1 had only *T. spiralis*, and 2 had mixed infections of *T. pseudospiralis* and *T. spiralis.*

## Methods

**Figure 1:**
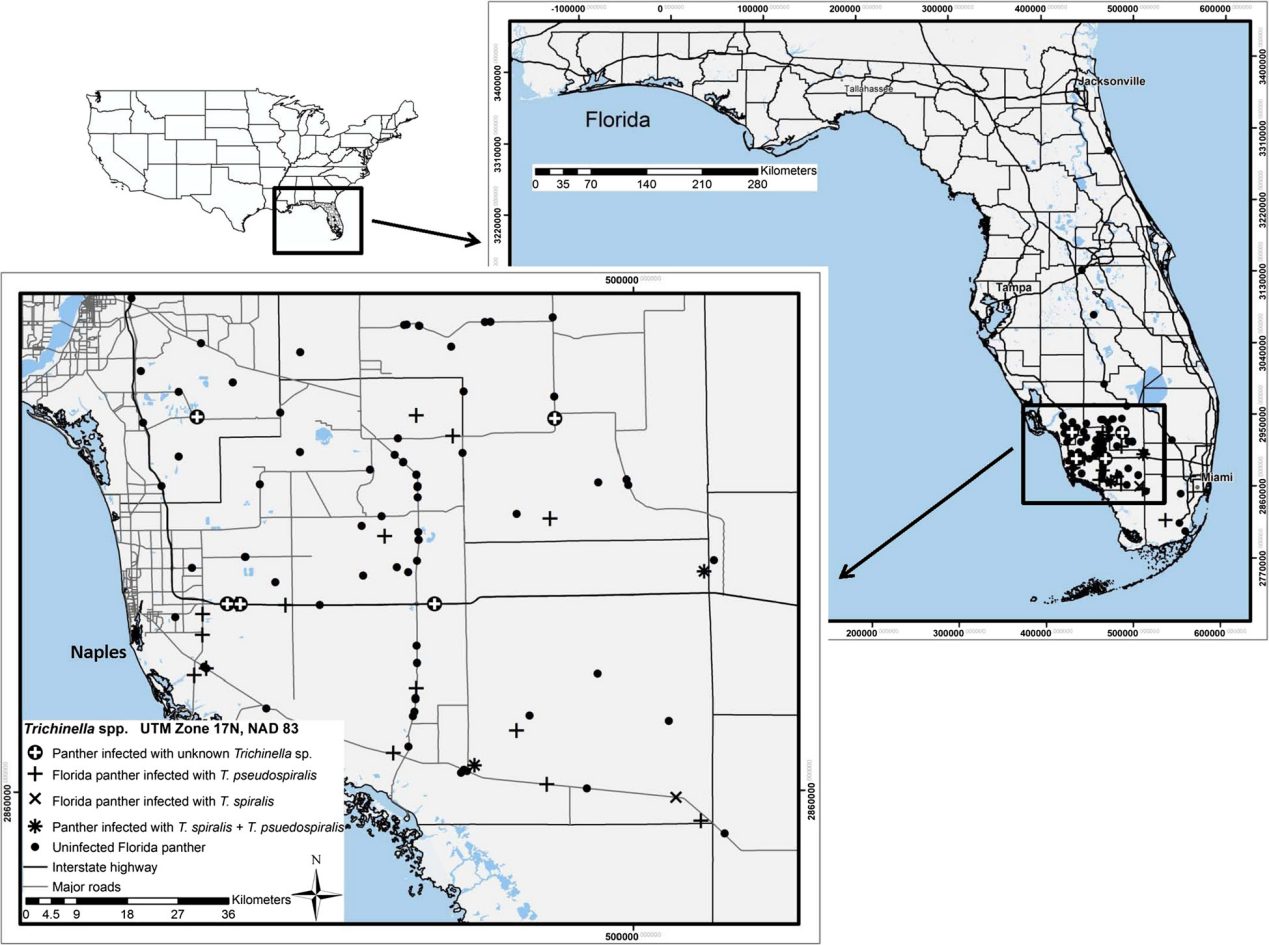
**Locations where Florida panthers (** ***Puma concolor coryi*** **) were collected and where** ***Trichinella pseudospiralis*** **and** ***Trichinella spiralis*** **were found from July 1999 until January 2011.**

As part of ongoing studies to assess mortality of Florida panthers, tongues from dead panthers were collected at necropsy from July 1999–January 2011. Samples originated from an area ranging from 25°08′–30°20′ North and 80°03′–82°12′ West (Figure 1). Sex, age class (kitten [≤2 months, still in the den]; dependent juveniles [≤1.1 years, out of the den but still with dam]; adult [≥1.2 years]), and collection locations were recorded in Universal Transverse Mercator (UTM) for each panther. Tongues in varying degrees of decomposition (panther dead a few hours to a day or more) were frozen (−20°C) until they were shipped on ice packs to the Center for Veterinary Health Sciences at Oklahoma State University (OSU) in January 2011. Once the tongues arrived at OSU, they were still frozen and, subsequently, were placed at −20°C until they were processed to determine infection with *Trichinella*.

### *Trichinella* spp. detection

Panther tongues were tested for infection with *Trichinella* spp. by artificial digestion [9]. Approximately 5.0 g of tissue were weighed (to the nearest 0.1 gram) and homogenized with a Polytron (Kinematica GmbH, Kriens-Luzern, Switzerland). Homogenized samples were mixed with 10 mL of artificial digestive fluid (1% pepsin 1:10,000 IU and 1% hydrochloric acid) per 1.0 g of tissue. Digests were mixed vigorously on magnetic stir plates at 37°C for 30 minutes. After 30 minutes, digests were immediately cooled on ice and allowed to settle for 20 minutes [10]. Sediment was washed with tap water 3 to 5 times, depending on the amount of cellular debris, and samples were scanned for larvae using a stereomicroscope at 40× magnification. Results were recorded as the number of *Trichinella* sp. larvae per g (LPG) of tissue digested.

### Molecular characterization of *Trichinella* spp. Larvae

*Trichinella* spp. larvae recovered by artificial tissue digestion from panthers were washed in saline, preserved in absolute ethyl alcohol, and submitted to the International *Trichinella* Reference Center (ITRC, www.iss.it/site/Trichinella/) in Rome, Italy for genotyping. Individual *Trichinella* spp. larvae were identified by multiplex PCR analysis following the protocol described by Zarlenga *et al.* [11] and modified by Pozio and La Rosa [10]. Briefly, DNA was extracted from 10 individual worms of each isolate. PCR was performed using ExTaq DNA polymerase (Takara) in 50 ml containing 1.5 mM MgCl_2_, 200 mM dNTPs, 50 pmol of each primer and 0.5 unit of ExTaq DNA polymerase. The PCR-amplified fragments from purified DNA were visualised by agarose gel electrophoresis (2.0% standard agarose). Single *Trichinella* sp. larvae from one reference strain (ITRC code) for each taxa circulating in North America, were used for comparison: *T. spiralis* (ISS003), *T. nativa* (ISS010), *T. pseudospiralis* (ISS470), *T. murrelli* (ISS035), and *Trichinella* genotype T6 (ISS040).

### Statistics

The prevalence of *Trichinella* spp. infection in Florida panthers was calculated according to Bush *et al*. [12]. 95% confidence intervals were calculated according to Sterne’s exact method [13] using Quantitative Parasitology 3.0 [14]. Comparisons of the prevalence of *Trichinella* spp. between sex and age class of panthers and year collected were done using Chi-square or Fisher’s Exact tests [15]. Mann–Whitney Rank Sum tests [15] were used to compare *Trichinella* sp. LPG between sex and age of infected panthers and year collected. Chi-square, Fisher’s Exact and Mann–Whitney Rank Sum tests were performed using SigmaPlot 12.5 statistical software (Systat Software Inc, San Jose, California, United States).

### Ethics

Tongues from Florida panthers were collected opportunistically at necropsy. The panthers were being necropsied in part to determine the cause of death following a natural mortality event. As the panthers, while alive, were not manipulated for the purposes of the current research, ethical approval was not necessary.

## Results

### *Trichinella* Larvae detection

Tongues from 112 Florida panthers collected across southern Florida (Figure 1) were tested for infection with *Trichinella* spp. (Table 1). The prevalence of *Trichinella* spp. infection (95% confidence interval) was 21.4% (14.6%–30.3%). Significantly more (*X*^2^ = 3.977, df = 1, P = 0.046) male panthers were infected with *Trichinella* spp. 28.1% (18.2%–40.6%) than females 12.5% (5.6%–24.8%). Infection of *Trichinella* spp. was not detected in 4 kittens sampled. Of 18 dependent juvenile Florida panthers tested for infection with *Trichinella* spp., 4 (all males) were infected (22.2%; 8.0%–47.1%). The prevalence of *Trichinella* spp. in adult panthers was 22.2% (14.5%–32.1%) and although greater, was not statistically discernible (*X*^2^ = 1.131, df = 2, P = 0.568) from other age classes. Prevalence of *Trichinella* spp. infection was not statistically impacted by the year samples were collected (Table 2).

**Table 1:** **Number infected and median** ***Trichinella*** **spp. larvae per g (LPG) in tongues of Florida panthers determined by tissue digestion according to sex and age**

**Sex**	**Age**	**Number of samples**	**Number infected (%; 95% CI)**	**Median LPG (SE; min–max)**
Male	Kitten	0	0 (0.0%; 0.0–0.0)	0.0 (0.0; 0.0–0.0)
	Dependent	10	4 (40.0%; 15.0%–70.9%)	13.4 (80.1; 0.4–329.0)
	Adult	54	14 (25.9%; 15.5%–39.7%)	0.9 (0.5; 0.2– 7.2)
Female	Kitten	4	0 (0.0%; 0.0%–0.0%)	0.0 (0.0; 0.0–0.0)
	Dependent	8	0 (0.0%; 0.0%–0.0%)	0.0 (0.0; 0.0–0.0)
	Adult	36	6 (16.7%; 7.5%–32.0%)	0.9 (2.1; 0.2–11.6)
Total		112	24 (21.4%; 14.6%–30.3%)	1.0 (13.6; 0.2–329.0)

**Table 2:** **Number infected and median** ***Trichinella*** **spp. larvae per g (LPG) in tongues of Florida panthers determined by tissue digestion according to year collected**

**Year sampled**	**Number of samples**	**Number infected (%; 95% CI)**	**Median LPG (SE; min–max)**
2000	1	0 (0.0%; 0.0%–0.0%)	0.0 (0.0; 0.0–0.0)
2001	8	1 (12.5%; 0.6%–50.0%)	1.2 (NA; 1.2–1.2)
2002	7	0 (0.0%; 0.0%–0.0%)	0.0 (0.0; 0.0–0.0)
2004	14	4 (28.6%; 10.4%–57.4%)	1.2 (0.6; 0.2–2.4)
2005	10	4 (40.0%; 15.0%–70.9%)	5.1 (81.3; 0.8–329.0)
2006	12	4 (33.3%; 12.3%–63.0%)	1.8 (5.5; 0.2–23.0)
2007	16	3 (18.8%; 5.3%–43.6%)	7.2 (3.3; 0.2–11.6)
2008	9	2 (22.2%; 4.1%–55.8%)	0.8 (0.0; 0.8–0.8)
2009	14	0 (0.0%; 0.0%–0.0%)	0.0 (0.0; 0.0–0.0)
2010	19	5 (26.3%; 11.0%–50.0%)	0.4 (0.7; 0.2–3.8)
2011	2	1 (50.0%; 2.5%–97.5%)	0.5 (NA; 0.5–0.5)
**Total**	**112**	**24**	**1.0 (13.6; 0.2–329.0)**

The median LPG (SE; range) of *Trichinella* spp. observed in tongues according to sex and age classes of Florida panthers are presented in Table 1. Median *Trichinella* spp. LPG was 1.0 (13.6; 0.2–329.0). Statistically distinguishable differences in *Trichinella* spp. LPG were not detected among sex (U = 52.5, P = 0.947) nor age class (U = 17.0, P = 0.081) of panthers. Median *Trichinella* spp. LPG was not statistically noticeable among years samples were collected.

### Molecular identification

Amplifiable DNA from *Trichinella* spp. first-stage larvae was obtained from 19 of the 24 infected Florida panthers. No PCR amplification was obtained from larvae of 5 isolates; probably due to DNA degradation from multiple freeze-thaw events. Banding patterns from multiplex PCR amplifications showed 16 Florida panthers infected with *T. pseudospiralis* (ITRC codes: ISS5109, ISS5111, ISS5112, ISS5113, ISS5114, ISS5115, ISS5116, ISS5117, ISS5118, ISS5119, ISS5120, ISS5196, ISS5197, ISS5201, ISS5202, and ISS5203), 2 co-infected with *T. pseudospiralis* and *T. spiralis* (ISS5198 and ISS5199), and 1 infected with *T. spiralis* (ISS5200). The expansion segment five of the large subunit ribosomal DNA of *T. pseudospiralis* larvae displayed a band pattern of 340 bp.

## Discussion

Previous examination of tongues and/or diaphragms collected from 7 Florida panthers between March 1978 to December 1983 using tissue squash or pepsin-HCl digestion showed 4 (57.1%) were infected with *Trichinella* spp*.* [8]*.*Odds ratio analysis showed that Florida panthers sampled by Forrester *et al*. were 4.9 times more likely to be infected with *Trichinella* spp. than those in the current study. Differences in the prevalence of *Trichinella* spp. infection detected between the two studies are likely explained by several factors. We examined 16 times more Florida panthers from a larger geographical area over a longer period of time. Interestingly, this difference may also be attributed to genetic introgression of Florida panthers in 1995 when 8 female Texas pumas (*P. c. cougar*) were released into the population to increase depleted genetic diversity [3].

Infection of *Trichinella* spp. has been documented in other wildlife from Florida. In 1962, Scholtens and Norman [16] sampled diaphragms from 224 fur-bearing animals collected in Marion County (north-central), Florida. *Trichinella* spp. larvae were detected by artificial digestion in 1 of 17 (5.9%) foxes (*Urocyon cinereoargenteus* and *Vulpes* [*fulva*] *vulpes*), 3 of 65 (4.6%) opossums (*Didelphis* [*marsupialis*] *virginiana*), 4 of 109 (3.7%) raccoons, and 1 of 22 (4.5%) skunks (*Mephitis mephitis* and *Spilogale putorius*) [16]. Eleven bobcats (*Lynx rufus*) were sampled; however, *Trichinella* sp. was not detected in any of the bobcats. Because the Scholtens and Norman survey was conducted before the advent of current taxonomy which identifies 12 taxa in this genus [2], it is uncertain whether these wild animals were actually infected with *T. spiralis* or a different species. Nonetheless, the routine occurrence of *Trichinella* spp. in Florida panthers and some of their prey animals suggests it is common for this endangered wild felid to be infected.

The presence of *T. pseudospiralis* and *T. spiralis* in Florida panthers and the absence *T. murrelli* was surprising. *Trichinella murrelli* is the predominant species that infects wild carnivores, but not swine, in temperate regions of the US [17] and suggests that the main source of *Trichinella* spp. infections for Florida panthers were wild pigs. This is in agreement with the diet of Florida panthers in which wild pigs can represent ~42% [6] of their prey and with the detection of anti-*Trichinella* IgG in 5.6% of wild pigs from Florida [18]. In Europe, *T. pseudospiralis* is common in wild pigs and carnivores even if its prevalence is much lower than that of *T. spiralis* and *T. britovi* (www.iss.it/Trichinella/).

Infection of *T. pseudospiralis* in North American animals had been documented only three times previously: in a black vulture (*Coragyps atratus*) from Alabama in 1985 [19], in a wild pig from Texas in 2005, and in a mountain lion (*P. c. cougar*) from British Columbia in 2010 [20]. Prior to these confirmed cases, *T. pseudospiralis* was suspected in a Cooper’s hawk (*Accipiter cooperi*) from California in 1982 [21,22], a great horned owl (*Bubo virginianus*) from Iowa in 1959 [23], and a pomarine jaeger (*Stercorarius pomarinus*) from Alaska in 1956 [24]. The band pattern of 340 bp for *T. pseudospiralis* is a hallmark identifying isolates which belong to the North American population of this parasite [25]. The common occurrence of *T. pseudospiralis* in Florida panthers reported in the current study suggests it is likely these wild felids are a key mammalian species in the transmission and ecology of this parasite in Florida. The current study comprises the first report of *T. pseudospiralis* in Florida.

*Trichinella spiralis* is adapted to and most commonly occurs in domestic and wild pigs [26]. Today the occurrence of *T. spiralis* in the US is rare [2]. It is sporadically reported in free-ranging pigs and poorly managed domestic swine in the US [18,26]. *Trichinella spiralis* spills over into wildlife when there is a current or historic occurrence of the parasite in pigs [18,27,28]. For example, on a poorly managed pig farm in Maryland, the overall prevalence of *T. spiralis* in adjacent raccoon and opossum populations was 41% (7 of 17) 6 months after pigs had been depopulated from the farm [27].The prevalence of *T. spiralis* dropped to 10% (1 of 10) one year after pigs were depopulated and was undetectable (0 of 15) in wild scavengers 18 months after pigs were removed from the farm [27]. A study conducted in 1993 demonstrated that 0.3% (4 of 1294) of domestic and 2.8% (5 of 179) of wild pigs in Florida had anti-*Trichinella* IgG in their sera [29]. However, the specificity of the serological test used at that time can be questioned [30].

Mixed infections of *T. pseudospiralis* and *T. spiralis* had been documented in a wild pig from Germany [31], a raccoon dog (*Nyctereutes procyonoides*) and a red fox from Germany, a red fox from Bulgaria, and a domestic pig from Bosnia-Herzegovina (www.iss.it/Trichinella/). In the current study, mixed infections of *T. pseudospiralis* and *T. spiralis* were documented in two Florida panthers. It is likely these two panthers became infected with *T. pseudospiralis* and *T. spiralis* on separate occasions but exactly how they became infected is unknown. Laboratory studies demonstrated that immunity to re-infection is influenced by the host immune response, the *Trichinella* species of first exposure, and the worm burden [32].

The prevalence (21.4%) of *T. pseudospiralis* in Florida panthers is, to our knowledge, one of the highest of this species detected in a single host species in the world reported to date. A higher prevalence of *T. pseudospiralis* (30%; 46 of 153) was detected in Tasmanian devils (*Sarcophilus harrisii*) [33] from Tasmania. However, the epidemiology of *T. pseudospiralis* in Florida and Tasmania should be dissimilar due to different environmental and ecological patterns including the presence of wild pigs in Florida but not Tasmania. The different transmission patterns suggest a high plasticity of *T. pseudospiralis*, which is one of the most widespread parasitic nematodes circulating in wild animals.

## Conclusions

*Trichinella pseudospiralis* seems to be a common zoonotic parasite of the entozoic habitat of the Florida panthers, whereas *T. spiralis* seems to be less prevalent. Wild pigs are likely the main source of *T. pseudospiralis* and *T. spiralis* to Florida panthers as these swine can be host to both parasites and are a main food source for the cats. The prevalence (21.4%) of *T. pseudospiralis* in Florida panthers is one of the highest of this parasite detected in the world. The high prevalence of *T. pseudospiralis* in Florida panthers, in combination with reports of this parasite in a variety of other mammals and birds from distinct geographic locations, suggests *T. pseudospiralis* is one of the most widespread nematodes in wild animals.
